# Anti-Inflammatory Effect of Geniposide on Regulating the Functions of Rheumatoid Arthritis Synovial Fibroblasts via Inhibiting Sphingosine-1-Phosphate Receptors1/3 Coupling Gαi/Gαs Conversion

**DOI:** 10.3389/fphar.2020.584176

**Published:** 2020-12-08

**Authors:** Rong-hui Wang, Xue-jing Dai, Hong Wu, Meng-die Wang, Ran Deng, Yan Wang, Yan-hong Bu, Ming-hui Sun, Heng Zhang

**Affiliations:** ^1^ Key Laboratory of Xin’an Medicine, Ministry of Education, Hefei, China; ^2^ College of Pharmacy, Anhui University of Chinese Medicine, Hefei, China; ^3^ Anhui Province Key Laboratory of Research and Development of Chinese Medicine, Hefei, China; ^4^ Anhui Province Key Laboratory of Chinese Medicinal Formula, Hefei, China

**Keywords:** gαi-gαs conversion, sphingosine-1-phosphate receptor 1/3, sphingosine-1-phosphate, geniposide, fibroblast-like synoviocytes, rheumatoid arthritis

## Abstract

The activated Gα protein subunit (Gαs) and the inhibitory Gα protein subunit (Gαi) are involved in the signal transduction of G protein coupled receptors (GPCRs). Moreover, the conversion of Gαi/Gαs can couple with sphingosine-1-phosphate receptors (S1PRs) and have a critical role in rheumatoid arthritis (RA). Through binding to S1PRs, sphingosine-1-phosphate (S1P) leads to activation of the pro-inflammatory signaling in rheumatoid arthritis synovial fibroblasts (RASFs). Geniposide (GE) can alleviate RASFs dysfunctions to against RA. However, its underlying mechanism of action in RA has not been elucidated so far. This study aimed to investigate whether GE could regulate the biological functions of MH7A cells by inhibiting S1PR1/3 coupling Gαi/Gαs conversion. We use RASFs cell line, namely MH7A cells, which were obtained from the patient with RA and considered to be the main effector cells in RA. The cells were stimulated with S1P (5 μmol/L) and then were treated with or without different inhibitors: Gαi inhibitor pertussis toxin (0.1 μg/mL), S1PR1/3 inhibitor VPC 23019 (5 μmol/L), Gαs activator cholera toxin (1 μg/mL) and GE (25, 50, and 100 μmol/L) for 24 h. The results showed that GE may inhibit the abnormal proliferation, migration and invasion by inhibiting the S1P-S1PR1/3 signaling pathway and activating Gαs or inhibiting Gαi protein in MH7A cells. Additionally, GE could inhibit the release of inflammatory factors and suppress the expression of cAMP, which is the key factor of the conversion of Gαi and Gαs. GE could also restore the dynamic balance of Gαi and Gαs by suppressing S1PR1/3 and inhibiting Gαi/Gαs conversion, in a manner, we demonstrated that GE inhibited the activation of Gα downstream ERK protein as well. Taken together, our results indicated that down-regulation of S1PR1/3-Gαi/Gαs conversion may play a critical role in the effects of GE on RA and GE could be an effective therapeutic agent for RA.

## Introduction

Rheumatoid arthritis (RA) is a chronic, inflammatory and autoimmune disease whose etiology is complex and unclear. It is distinguished mainly by the abnormal hyperplasia of synovial tissue, infiltration of inflammatory immune cells, angiogenesis and pannus formation (Firestein and McInnes, 2017; Guo et al., 2018). Generally, its main clinical manifestation is the destruction of articular cartilage, even causing joint deformity and loss of labor ability (Aletaha and Smolen, 2018). Fibroblast-like synoviocytes (FLSs) have been considered to be the main effector cells in RA, and their biological functions changes play a crucial role in the course of RA (Bustamante et al., 2017; Doody et al., 2017). Abnormal proliferating FLSs can stimulate the expression of a large number of inflammatory factors and mediators, such as sphingosine-1-phosphate (S1P), Interleukin (IL)-1β, IL-6, cyclic adenosine phosphate (cAMP), transforming growth factor-β1(TGF-β1), prostaglandin E2 (PGE2) and so on, leading to infiltration and hypoxia of the inflammatory cells. Finally, they lead to an imbalance of synovial microenvironment and even induce the body’s immune dysfunctions (Fearon et al., 2016; Yang et al., 2016; Yang et al., 2019). Therefore, inhibiting the dysfunctions of FLSs has become a focus of clinical treatment of RA.

G protein coupled receptors (GPCRs) are a class of highly conserved membrane protein families that play a significant role in the regulation of immune responses (Lu and Cyster, 2019). The G protein is composed of α, β and γ subunits. Gα protein can be further divided into Gαi, Gαs, Gαt, Gαo, Gαq, Gαz and Gα12/13 subtypes (Seven et al., 2020). Gα protein binds to downstream intracellular kinases, including extracellular signal-regulated kinase 1/2 (ERK1/2), phosphatidylinositol 3-kinase (PI3K), p38 mitogen-activated protein kinase (p38 MAPK), Rho and Ras (Yu et al., 2018). It has been proved that these proteins are closely related to the regulation of rheumatoid arthritis synovial fibroblasts (RASFs) proliferation, apoptosis, differentiation and other biological functions (Vázquez-Victorio et al., 2016; Han et al., 2020). In addition, the inhibitory Gα protein subunit (Gαi) and activated Gα protein subunit (Gαs) can reduce the production of second messenger cAMP by regulating the activation of adenylate cyclase (AC) and mediate downstream signaling pathways to regulate inflammation reaction (Jia et al., 2014). Gαi and Gαs also regulate the abnormal proliferation of RASFs by mediating cAMP secretion (de Castro Abrantes et al., 2019). It has been confirmed that increase the expression level of Gαi and decrease the expression level of Gαs in FLSs from rats with collagen-induced arthritis, resulting in a decrease in AC activity and a decrease in cAMP production, which in turn causes abnormal synovial cells proliferation (Han, et al., 2020). To sum up, the dynamic balance and conversion of Gαi/Gαs are crucial in RA.

S1P is a biologically active lysophosphatidic, which is catalyzed by sphingomyelin under the action of nerve phospholipase, ceramide enzyme and sphingosine kinase (Ebenezer et al., 2017). The previous studies have been proved that there is a large amount of S1P in the synovial fluid and blood in adjuvant arthritis (AA) rats (Xuejing et al., 2019). Additionally, the concentration of S1P in the synovial fluid of RA patients is significantly higher than that in the blood (Zhao et al., 2019). S1P also induces an inflammatory response by promoting the release of inflammatory factors in synoviocytes and endothelial cells (Hu et al., 2020). S1P works in conjunction with five GPCRs, namely S1PR1-5. S1PR1, S1PR2 and S1PR3 commonly express in most tissues, S1PR4 only express in lymphoid tissues and lungs and S1PR5 mainly express in the central nervous system (Spiegel and Milstien, 2011; Mohammed and Harikumar, 2017). Additionally, S1PR1 and S1PR3 mainly involve in the physiological and responses of FLSs, while the role of S1PR2 is not obvious (Zhao et al., 2008). S1PRs couple with distinct G-proteins regulate diverse physiological functions. S1PRs couple with Gα can regulate downstream signaling pathways such as MAPKs, PI3K/AKT and so on. It can also mediate cells proliferation and differentiation and regulate the secretion of inflammatory factors (Tsai and Han, 2016; Zhu et al., 2018). S1PR1 expression is closely related to RA synovial hyperplasia and angiogenesis, especially plays an important role in RA synovial cells proliferation and regulates cAMP release (Jin et al., 2016). S1PR3 is more highly expressed in the synovium of arthritis (Inoue et al., 2019). S1PR1/3 is mainly involved in the inflammatory response, promoting cells angiogenesis, differentiation and proliferation (Hsu et al., 2015; Chen et al., 2020). S1P can stimulate Gαi and Gαs protein-dependent signaling expression and regulate the level of cAMP expression (Lagadari et al., 2009). S1P/S1PRs/Gα-mediated signaling pathway plays a central role in inflammation. Therefore, S1P may play a unique role in RA through binding to S1PR and coupling with Gαi/Gαs-mediated inflammation. However, whether S1P regulates the biological functions of RASFs through S1PR1/3 mediating Gαi/Gαs conversion has not been reported and the effects of GE on regulating the biological functions of RASFs are not veiled.

Geniposide (GE) is an iridoid compound extracted from *Gardenia jasminoides Ellis*, which has been shown to have anti-inflammatory and other pharmacological effects (Fu et al., 2012; Wang et al., 2018). The previous research has proved that GE can reduce the level of inflammatory mediators in AA rats (Deng et al., 2018). Furthermore, we have revealed that GE may reduce the level of inflammatory cytokines and maintain dynamic balance of pro/anti-inflammatory cytokines in RASFs by increasing the activation of inflammatory signaling pathways such as Raf/MEK/ERK1/2, RhoA/p38MAPK/NF-κB/F-actin pathways (Deng et al., 2018; Li et al., 2018). In addition, GE can block Sphk1/S1P/S1PR1 pathway hyperactivation, inhibit the abnormal proliferation, invasion and migration of RASFs (Sun et al., 2020), and hence it plays a crucial role in the treatment of RA. To sum up, all these studies suggest that GE regulates the inflammation and biological functions of RASFs.

In the present study, the effectiveness of GE in S1PR1/3-Gαi/Gαs pathway was investigated in MH7A cells by S1P-induced inflammation model. We found that S1P leads to the imbalance of Gαi/Gαs through binding to S1PR1/3. In addition, we confirmed that GE could regulate Gαi/Gαs conversion via inhibiting the expression of S1PR1/3 and regulate downstream inflammatory signaling pathways. Thus, GE is involved in regulating the biological functions of MH7A cells and has a therapeutic effect on RA.

## Materials and Methods

### Materials and Reagents

GE was provided by the National Institute for the Control of Pharmaceutical and Biological Products (Beijing, China). GE purity was about 98.0%; S1P was purchased by Toronto Research Chemicals Inc. (Toronto, Canada); The following protein inhibitor were applied: Gαs activator (cholera toxin, CTX) was obtained from Sigma (St. Louis, MO, United States); Gαi inhibitor (pertussis toxin, PTX) and S1PR1/3 inhibitor (VPC 23019) was obtained from Cayman (Ann Arbor, MI, United States); *p*-ERK1/2 and ERK1/2 rabbit monoclonal antibody and S1PR1/3 polyclonal antibodies were purchased from Abcam (Cambridge, MA, United States); The mouse monoclonal antibodies against Gαi, Gαs, Ki67 were obtained from Santa Cruz Biotechnology (Dallas, TX, United States); Dulbecco’s modified Eagle’s medium (DMEM) for MH7A cells culture was purchased from HyClone (Logan, UT, United States); Fetal bovine serum (FBS) were supplied from Serana (Coln, Germany).

### Culture of MH7A Cells

MH7A cells were obtained from fibroblast-like synoviocytes in patients with RA and were purchased from BeNa Culture Collection. The cells were cultured in DMEM medium containing 5% FBS and 1% penicillin-streptomycin solution with 5% CO_2_ at 37°C. In order to establish the inflammation model of MH7A cells, different concentrations of S1P (0, 0.1, 0.5, 1, 5, and 10 μmol/L) were added to the MH7A cells culture medium for different hours (6, 12, 24, 48, and 72 h). Finally, we determined the cells to be treated with S1P (5 μmol/L), with or without different inhibitors CTX (1 μg/mL), PTX (0.1 μg/mL), VPC 23019 (5 μmol/L) and GE (25, 50 and 100 μmol/L) for 24 h to investigate its effects on cells functions.

### Cell Proliferation Assay

The proliferation of MH7A cells were tested by cell counting kit 8 (Japanese colleagues Chemical, Japan). MH7A cells were counted and plated on 96 wells according to cell density of 5 × 10^3^ cells/well and cells were incubated in DMEM at 37°C for 24 h. Thereafter, the cells were treated with S1P (5 μmol/L), with or without different inhibitors, activator (CTX, PTX and VPC 23019) and GE (25, 50, and 100 μmol/L). After the treatment, the original medium was aspirated, and 100 μL of DMEM medium containing 10% CCK-8 was added to each well for 1.5 h. Check the absorbance was measured at 450 nm with a microplate reader (Molecular Devices, United States).

### Cell Migration Assay

The migration of MH7A cells were determined by Transwell plate. First of all, the cells were treated with S1P (5 μmol/L), with or without different inhibitors (CTX, PTX and VPC 23019) and GE (25, 50 and, 100 μmol/L) for 24 h at 37°C with 5% CO_2_. Then, MH7A cells were counted and plated on inner chamber of 24-well Transwell at a concentration of 2 × 10^5^ cells/mL. At the same time, 500 μL DMEM medium containing 10% FBS was added into the outer chamber, which were incubated for 24 h. After incubated, cells migrated on to the outer chamber. Fixed the cells in the outer chamber with 4% paraformaldehyde for 15 min. Then we add 500 μL crystal violet to outer chamber to fix the migrated cells for 25 min. Non-migrating MH7A cells on the upper surface were wipe off by a cotton swab. The results were observed and measured under the microscope (Olympus, Japan).

### Cell Invasion Assay

Transwell plate method was used to detect the invasion ability of MH7A cells. Matrigel glue was mixed with DMEM medium at a ratio of 1: 8 and placed in the inner chamber at 100 μL/well for 6 h. The next treatments were the same as the cell migration assay.

### Enzyme-Linked Immunosorbent Assay

MH7A cells were seeded in a 6-well culture plate at a cell density of 1 × 10^6^ cells/well. Then, cells were treated with S1P (5 μmol/L), with or without different inhibitors, activator (CTX, PTX and VPC 23019) and GE (25, 50, and 100 μmol/L) for 24 h. The supernatant of MH7A cells were collected and the concentrations of cAMP, PGE2, IL-1β, IL-6, IL-8, and TGF-β1 were measured with ELISA kits (J&L Biological, China) according to the manual testing.

### RNA Isolation and qRT-PCR

Quantitative real-time PCR was applied to measure the mRNA level of S1PR1/3, Gαi 1/2/3, Gαs and cAMP. MH7A cells were seeded in a 6-well culture plate at a cell density of 1 × 10^6^ cells/well. Total RNAs were obtained from MH7A after 24 h of incubation and achieved by TRIzol (Invitrogen, USA). The OD value at 260 and 280 nm was determined with a microplate reader to evaluate the integrity of RNAs. RNAs were reverse-transcribed into cDNA by cDNA reverse transcription kit according to the manufacturer’s recommendations. The reverse transcription procedure is as follows: 95°C for 10 min, 40 cycles of denaturation at 95°C for 10 min and 60°C for 35 s and annealing extension at 55°C for 30 s. qPCR was determined by using Premix Ex Taq SYBR-Green PCR (Takara, Germany) according to manufacturer’s recommendations on an ABI PRISM 7300 (Applied Biosystems, United States). The primers used were 5′- GAA​AAC​CAA​GAA​ATT​CCA​CCG​A-3′ (Forward) and 5′- TTT​CAG​CAT​TGT​GAT​ATA​GCG​C-3′ (Reverse) for S1PR1; 5′- AGT​GGT​TCA​TCG​TGT​TGG​CTG​TG-3′ (Forward) and 5′- GCT​GCT​ATT​GTT​GCT​GCT​GCT​TG-3′ (Reverse) for S1PR3; 5′- TCA​ACC​AAA​TTA​CAT​CCC​GAC​TC-3′ (Forward) and 5′- TCT​GAC​CTC​CAC​ATC​AAA​CAT-3′ (Reverse) for Gαi1; 5′- GAA​CGA​CCT​GGA​GCG​TAT​TGC-3′ (Forward) and 5′- GCT​GAC​CAC​CCA​CAT​CAA​ACA-3′ (Reverse) for Gαi2; 5′- TGG​GAC​GGC​TAA​AGA​TTG​ACT-3′ (Forward) and 5′- TGT​GTT​TCT​ACA​ATG​CCT​GTG​GTC-3′ (Reverse) for Gαi3; 5′- CAG​GGC​TCT​AGG​AAA​CAT​ATG​T-3′ (Forward) and 5′- TGC​CCA​GTA​ATT​TCA​CTA​AGG​T-3′ (Reverse) for Gαs; 5′- AGG​TCC​TCA​GCT​ACA​AGG​AAG-3′ (Forward) and 5′- TCT​TGA​AGT​CAC​AAT​CCT​CTG​GT-3′ (Reverse) for cAMP; 5′- GCA​CCG​TCA​AGG​CTG​AGA​AC-3′ (Forward) and 5′- TGG​TGA​AGA​CGC​CAG​TGG​A-3′ (Reverse) for GAPDH, The mRNA level of target gene was normalized to GAPDH and the results were calculated by 2^−ΔΔCt^ data analysis.

### Western Blot Analysis

Western Blot was applied to analysis the expression of S1PR1, S1PR3, Gαi, Gαs proteins; proliferation marker proteins: Ki67 and related proteins in downstream signaling pathways: ERK1/2, *p*-ERK1/2. MH7A cells were mixed with RIPA lysate (Beyotime Biotechnology, China) for 10 min on ice. The levels of proteins were determined by BCA protein assay kit (Beyotime Biotechnology, China). The protein samples were electrophoresed on 10% SDS/PAGE gels at 120 V for 60–80 min and then transferred to a PVDF membrane at 200 mA for 2 h. After the membrane transfer, the NC membrane were removed to a TBST incubator box containing 5% skimmed milk powder, which was blocked at 37°C for more than 2 h. The protein band was incubated the primary antibodies overnight at 4°C on a shaker. Then, transferred the protein band to the secondary antibodies and incubated at room temperature for 1.5 h. The levels of target proteins were normalized to GAPDH and the Image J software was used for analysis.

### Protein Co-Expression Experiment

MH7A cells were seeded in a 24-well culture plate with sterilization slide at a cell density of 1 × 10^4^ cells/well. After cultured for 24 h, we fixed the cells with 4% paraformaldehyde for 30 min at 4°C. Permeabilized the cells for 15 min with 500 μL of 0.5% TritonX-100 permeabilization solution. 500 μL 5% bovine serum albumin blocking solution was used to block cells at room temperature for 2 h. Then we incubated the cells overnight at 4°C with primary antibody mixture of S1PR1-Gαi, S1PR1-Gαs, S1PR3-Gαi and S1PR3-Gαs in a wet chamber. After that, the cells were washed by PBS and incubated at room temperature in the dark for 2 h with FITC-labeled goat anti-rabbit and Cy3-labeled goat anti-mouse secondary antibody. After washing again in PBS, DAPI stained the nucleus and incubated for 20 min. Finally, we took the slide upside down on a glass slide with a drop of anti-fluorescence quencher, fixed the slide with nail polish, observed the fluorescence under a laser confocal microscope and took the pictures.

### Statistical Analysis

All the experimental data were analyzed by using SPSS 23.0 statistical software, one-way analysis of variance (ANOVA) with post hoc contrasts by Student-Newman-Keuls test for multiple groups statistical analysis. *p* < 0.05 was considered as statistically significant difference and expressed as mean ± standard error of mean (SEM). Most statistical figures were performed by GraphPad Prism.

## Results

### Geniposide Inhibited MH7A Cells Proliferation, Migration and Invasion

The previous experiments proved that S1P existed in the synovium and blood of AA rats. S1P could increase the secretion of inflammatory factors and promote the abnormal proliferation of RASFs. We determined the concentration and duration of S1P modeling by CCK-8 proliferation experiment (Figure 2A). The results showed that MH7A cells proliferation was observably increased by S1P treatment at 5 μmol/L at 24 h. Additionally, to further determined the effects of GE on the biological functions of MH7A cells were regulated by the S1PR1/3-Gαi/Gαs conversion, cells were treated with GE and different inhibitors or activator.

**FIGURE 1:**
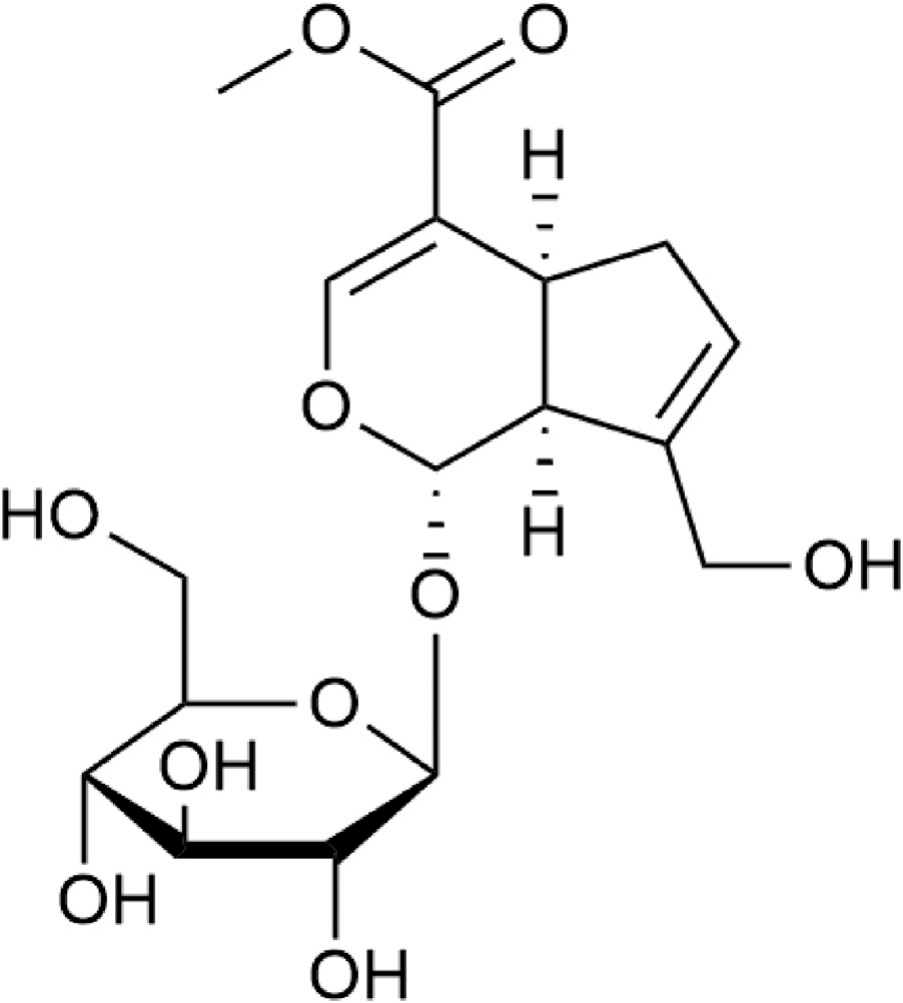
Chemical structure of geniposide.

**FIGURE 2:**
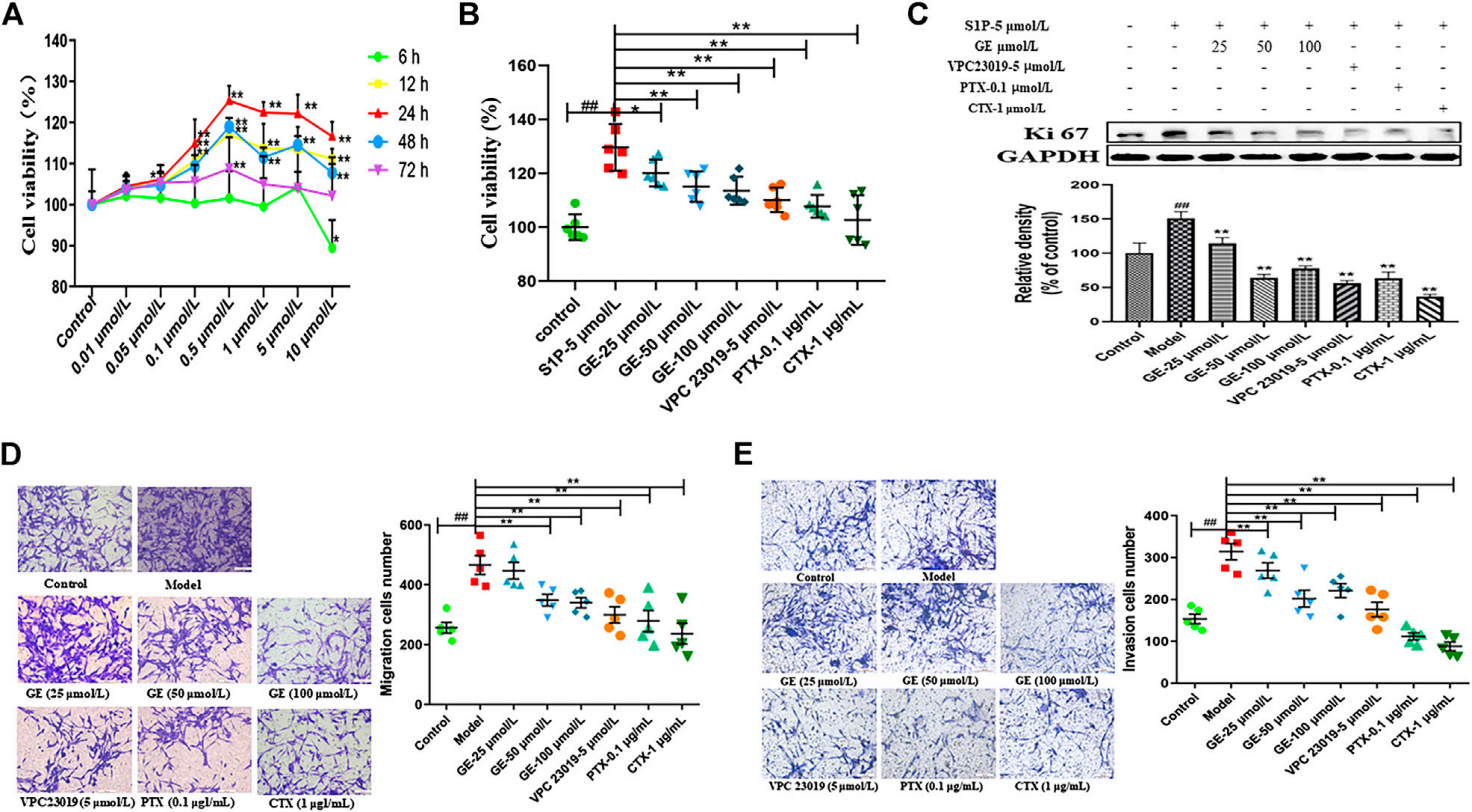
Effects of GE on proliferation, migration and invasion of MH7A cells induced by S1P. MH7A cells were treated with S1P (5 μmol/L) with or without different inhibitors Gαs activator CTX (1 μg/mL), Gαi inhibitor PTX (0.1 μg/mL), S1PR1/3 inhibitor VPC 23019 (5 μmol/L) and GE (25, 50 and 100 μmol/L) for 24 h. **(A)** Effect of S1P on the proliferation activity in MH7A was determined by CCK-8 kit (n = 6). **(B)** Effects of GE on the proliferation activity of MH7A induced by S1P was determined by CCK-8 kit (n = 6). **(C)** The proliferation protein level of Ki 67 was determined by Western blot (n = 3). **(D)** Effects of GE on the migration activity of MH7A was determined by Transwell (n = 5). **(E)** Effects of GE on the insion activity of MH7A was determined by Transwell (n = 5). Data are represented as mean ± SEM. ^*^ *p* < 0.05, ^**^ *p* < 0.01 versus control group; ^#^ *p* < 0.05, ^##^ *p* < 0.01 versus S1P (5 μmol/L) group.

The proliferation studies showed that compared with the normal group, the model group significantly promoted the MH7A proliferation activity, and the GE group could significantly reduce the S1P-induced MH7A proliferation activity in a dose-dependent manner. Compared with the model group, S1PR1/3 inhibitor VPC 23019, Gαi inhibitor PTX, and Gαs activator CTX also significantly inhibited MH7A proliferation activity (Figure 2B; *p* < 0.01). Meanwhile, the proliferation marker protein Ki67 was suppressed by the GE, inhibitors and activator (Figure 2C; *p* < 0.01). The results indicate that GE may inhibit the abnormal proliferation by inhibiting the S1P-S1PR1/3 signaling pathway and activating Gαs or inhibiting Gαi protein in MH7A cells.

The migration and invasion results were performed from the Transwell plate. As shown in Figures 2D, E, compared with the normal group, the migration ability of MH7A cells with S1P was significantly enhanced. In addition, the GE could significantly reduce the S1P-induced MH7A cells migration and invasion in a dose-dependent manner. The cells treated with S1PR1/3 inhibitor VPC 23019, Gαi inhibitor PTX, Gαs activator CTX can also significantly inhibit the migration and invasion ability of MH7A cells (*p* < 0.01). The studies showed that GE may inhibit the migration and invasion ability of MH7A cells by inhibiting the S1P-S1PR1/3 signaling pathway, activating Gαs or inhibiting Gαi protein.

GE inhibited S1PR1/3, Gαi expressions and up-regulated Gαs expression of MH7A cells induced by S1P.

**FIGURE 3:**
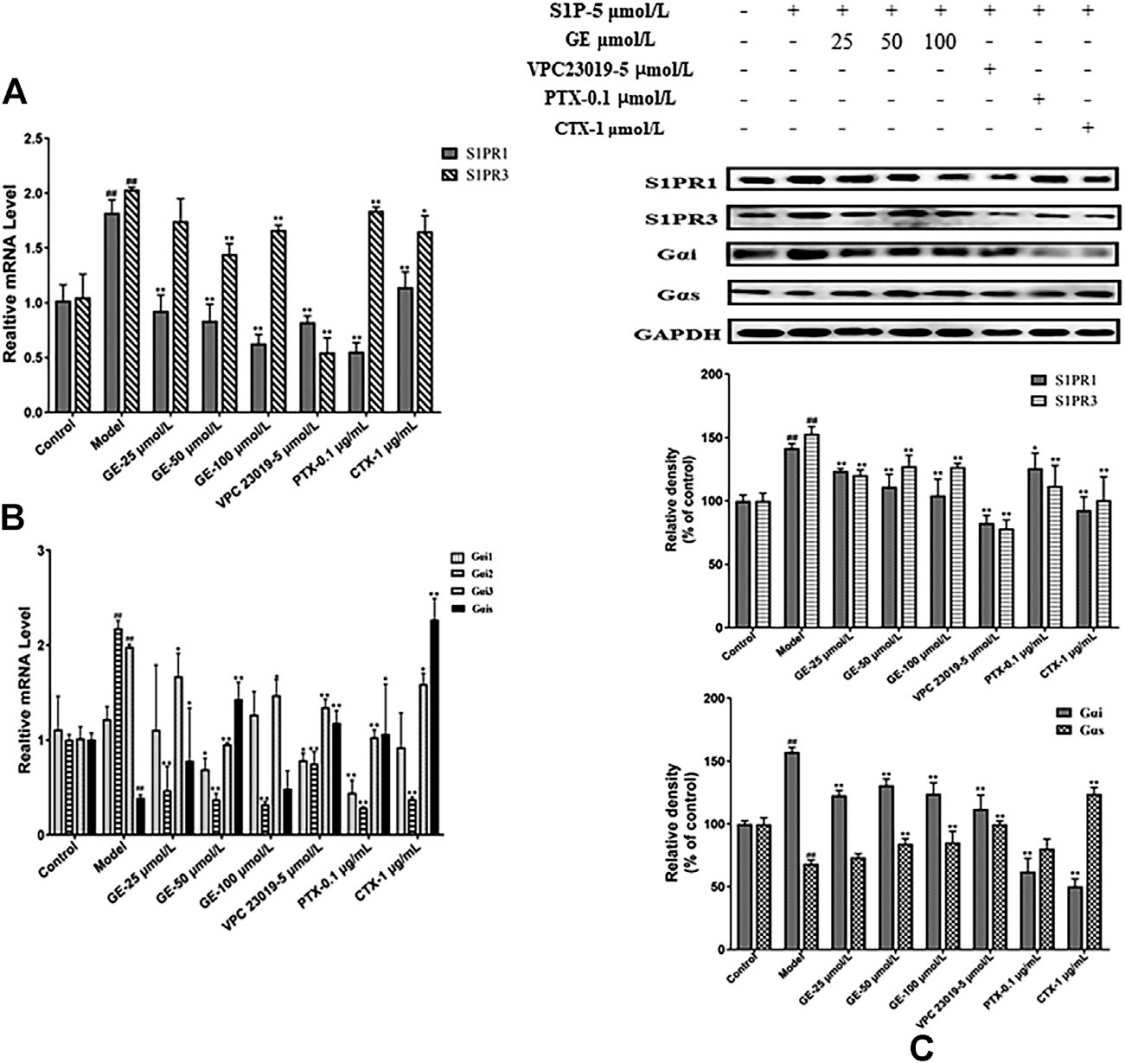
Effects of GE on the expression of S1PR1/3 and Gαi/Gαs. MH7A cells were treated with S1P (5 μmol/L) with or without different inhibitors Gαs activator CTX (1 μg/mL), Gαi inhibitor PTX (0.1 μg/mL), S1PR1/3 inhibitor VPC 23019 (5 μmol/L) and GE (25, 50 and 100 μmol/L) for 24 h. **(A)** Effects of GE on the expression of S1PR1 and S1PR3 mRNA in MH7A induced by S1P (n = 3). **(B)** mRNA expression of Gαi1/2/3 and Gαs was detected by qRT-PCR (n = 3). **(C)** The protein levels of S1PR1/3 and Gαi/Gαs were determined by Western blot analyses using specific antibodies for each protein (n = 3). Data are represented as mean ± SEM. ^*^ *p* < 0.05, ^**^ *p* < 0.01 versus control group; ^#^ *p* < 0.05, ^##^ *p* < 0.01 versus S1P (5 μmol/L) group.

From the above results, it was indicated that GE could regulated MH7A cells abnormal proliferation, migration and invasion preliminary. To further explore whether the effects of GE on biological functions of MH7A cells induced by S1P were regulated by the S1PR1/3 inhibitor VPC 23019, Gαi inhibitor PTX, and Gαs activator CTX, the cells were treated with S1P for 24 h and following treatment by GE and those inhibitors, activator. As seen in Figure 3A, the experimental results of qRT-PCR were shown that GE could significantly reduce S1PR1 and S1PR3 mRNA levels (*p* < 0.01). Additionally, Gαi2 and Gαi3 mRNA levels were significantly reduced and Gαs mRNA levels were increased by the treatment with GE (Figure 3B; *p* < 0.01). The results of cells treated with inhibitors and activator were the same as that of GE. The analysis results of western blot were shown in Figure 3C, as expected, GE, inhibitors and activator could downregulate the target S1PR1, S1PR3 and Gαi proteins, while upregulate Gαs protein (*p* < 0.01). Our data confirmed that GE is closely related to the S1PR1/3 coupled Gαi/Gαs conversion.

### Geniposide Suppressed cAMP Expression in MH7A Cells

**FIGURE 4:**
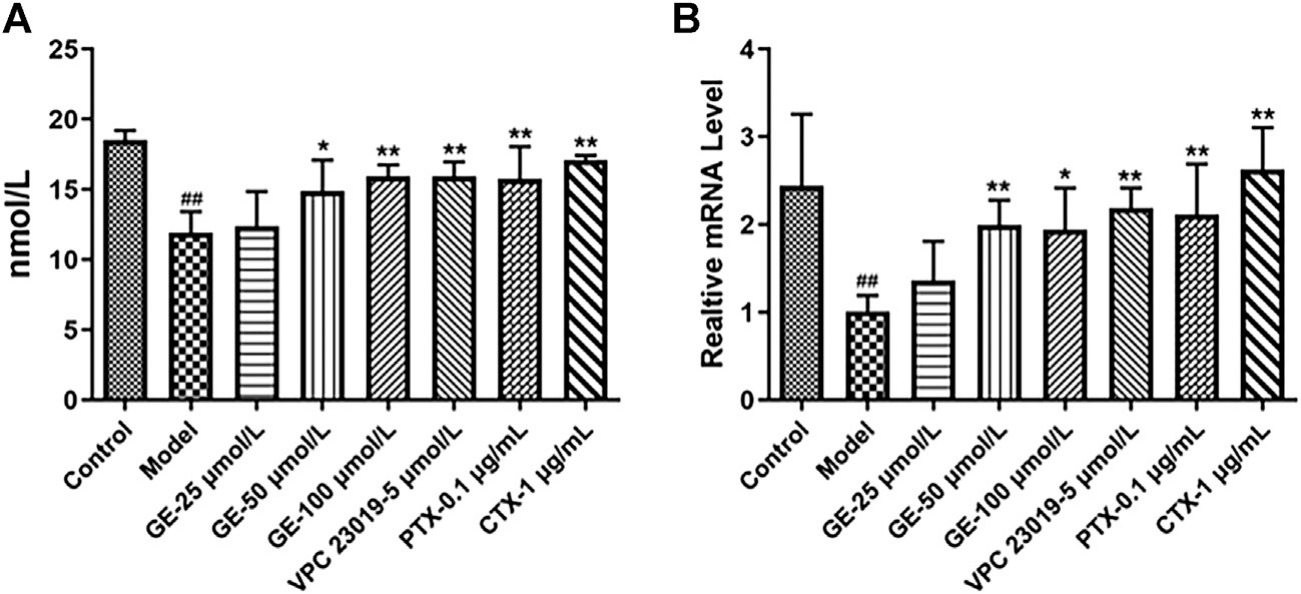
Effects of GE on the expression of cAMP in MH7A induce by S1P. MH7A cells were treated with S1P (5 μmol/L) with or without different inhibitors Gαs activator CTX (1 μg/mL), Gαi inhibitor PTX (0.1 μg/mL), S1PR1/3 inhibitor VPC 23019 (5 μmol/L) and GE (25, 50 and 100 μmol/L) for 24 h. **(A)** cAMP expression in MH7A was determined by ELISA analysis (n = 3). **(B)** mRNA expression of cAMP was detected by qRT-PCR (n = 3). Data are represented as mean ± SEM. ^*^ *p* < 0.05, ^**^ *p* < 0.01 versus control group; ^#^ *p* < 0.05, ^##^ *p* < 0.01 versus S1P (5 μmol/L) group.

cAMP mediates the downstream signaling pathway as a second messenger to regulate the inflammatory response. Gαi/Gαs can regulate the secretion of cAMP by regulating AC activation and mediate downstream signaling pathways to regulate the inflammatory response, while the combination of S1P and S1PRs could stimulate relevant downstream signaling pathways mediated by Gαi/Gαs. As seen in Figures 4A, B, GE could promote cAMP secretion. Under the action of S1PR1/3 inhibitor, Gαi inhibitor and Gαs activator, intracellular cAMP secretion level and mRNA expression level were significantly increased, while, S1P could inhibit the secretion of cAMP. These results indicated that GE could play an anti-inflammatory role by promoting the secretion of cAMP and that was related to the S1PR1/3-Gαi/Gαs signaling pathway.

### Geniposide Modulated Sphingosine-1-Phosphate-Induced Co-expression and Coupling of Sphingosine-1-Phosphate Receptors1/3 and Gαi/Gαs in MH7A Cells

**FIGURE 5:**
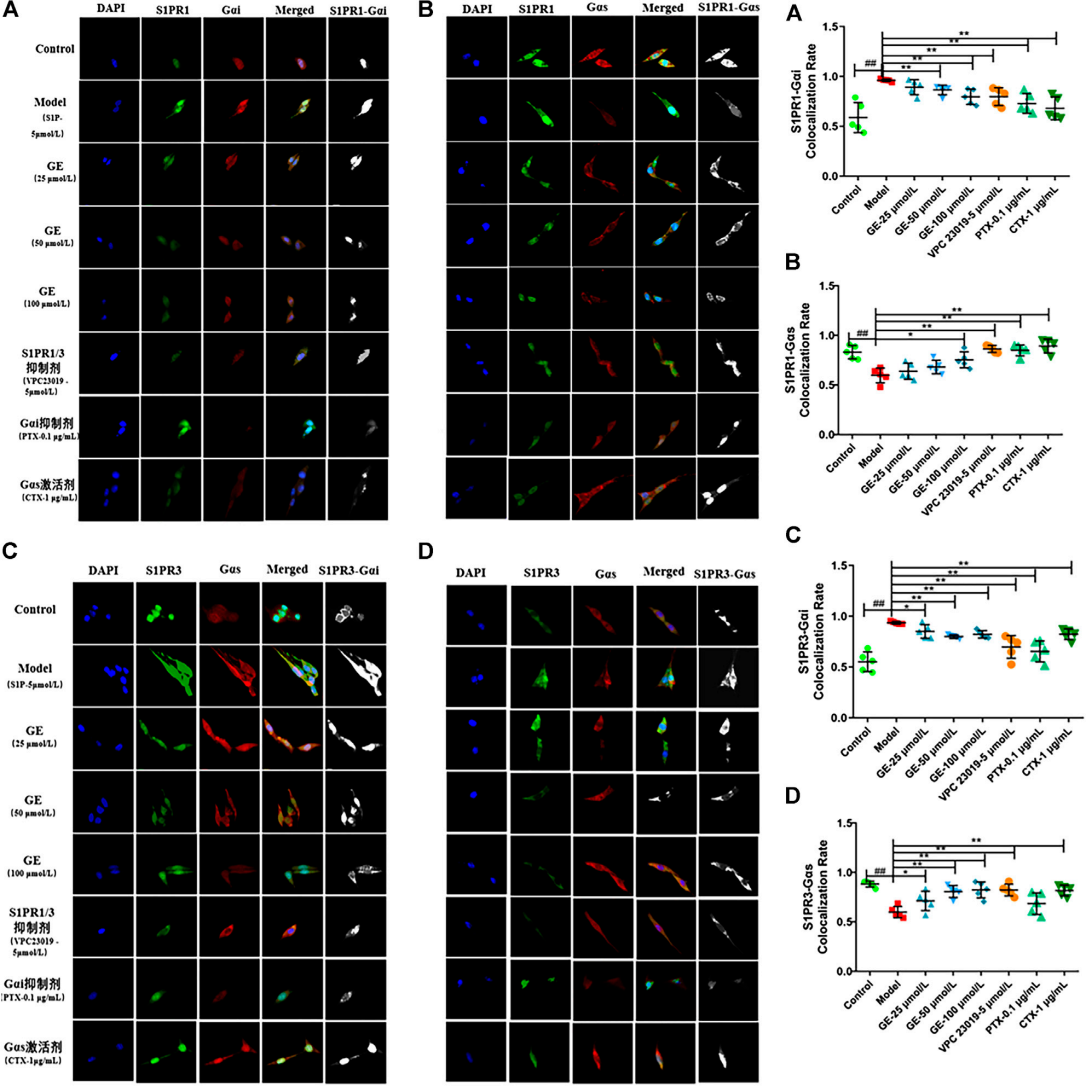
Effect of GE on S1PR1/3 co-expressing and coupling with Gαi/Gαs in MH7A induced by S1P. MH7A cells were treated with S1P (5 μmol/L) with or without different inhibitors Gαs activator CTX (1 μg/mL), Gαi inhibitor PTX (0.1 μg/mL), S1PR1/3 inhibitor VPC 23019 (5 μmol/L) and GE (25, 50 and 100 μmol/L) for 24 h. **(A)** Confocal imaging of S1PR1 and the Gαi in MH7A (n = 5). **(B)** Confocal imaging of S1PR1 and the Gαs in MH7A (n = 5). **(C)** Confocal imaging of S1PR3 and the Gαi in MH7A (n = 5). **(D)** Confocal imaging of S1PR3 and the Gαs in MH7A (n = 5). Data are represented as mean ± SEM. ^*^ *p* < 0.05, ^**^ *p* < 0.01 versus control group; ^#^ *p* < 0.05, ^##^ *p* < 0.01 versus S1P (5 μmol/L) group.

As can be seen from above, S1PR1/3 may couple with Gαi/Gαs conversion to cause inflammation. And we further explore the co-expression of S1PR1 with Gαi/Gαs, S1PR3 with Gαi/Gαs and the effects of GE. The confocal imaging showed that the co-expression of S1PR1 with Gαi was significantly enhanced under S1P induction, while in the GE group and the S1PR1/3 inhibitor VPC 23019 group, the co-expression of S1PR1 and Gαi was significantly decreased. Meanwhile, the co-expression of S1PR1 and Gαi was decreased to varying degrees under the action of Gαi inhibitor PTX and Gαs activator CTX. Similarly, the co-expression of S1PR1 and Gαs was enhanced compared with the S1P-induced inflammatory model group (Figures 5A, B; *p* < 0.01). Figures 5C, D showed that the coupling results of S1PR3 with Gαi/Gαs were the same as the coupling results of S1PR1 with Gαi/Gαs (*p* < 0.01). Those results were shown in fluorescence intensity and indicated that GE participates in the regulation of inflammation of MH7A cells by inhibiting S1PR1/3, Gαi and activating Gαs proteins.

### Geniposide Modulated Cytokine Secretion in MH7A Cells

**FIGURE 6:**
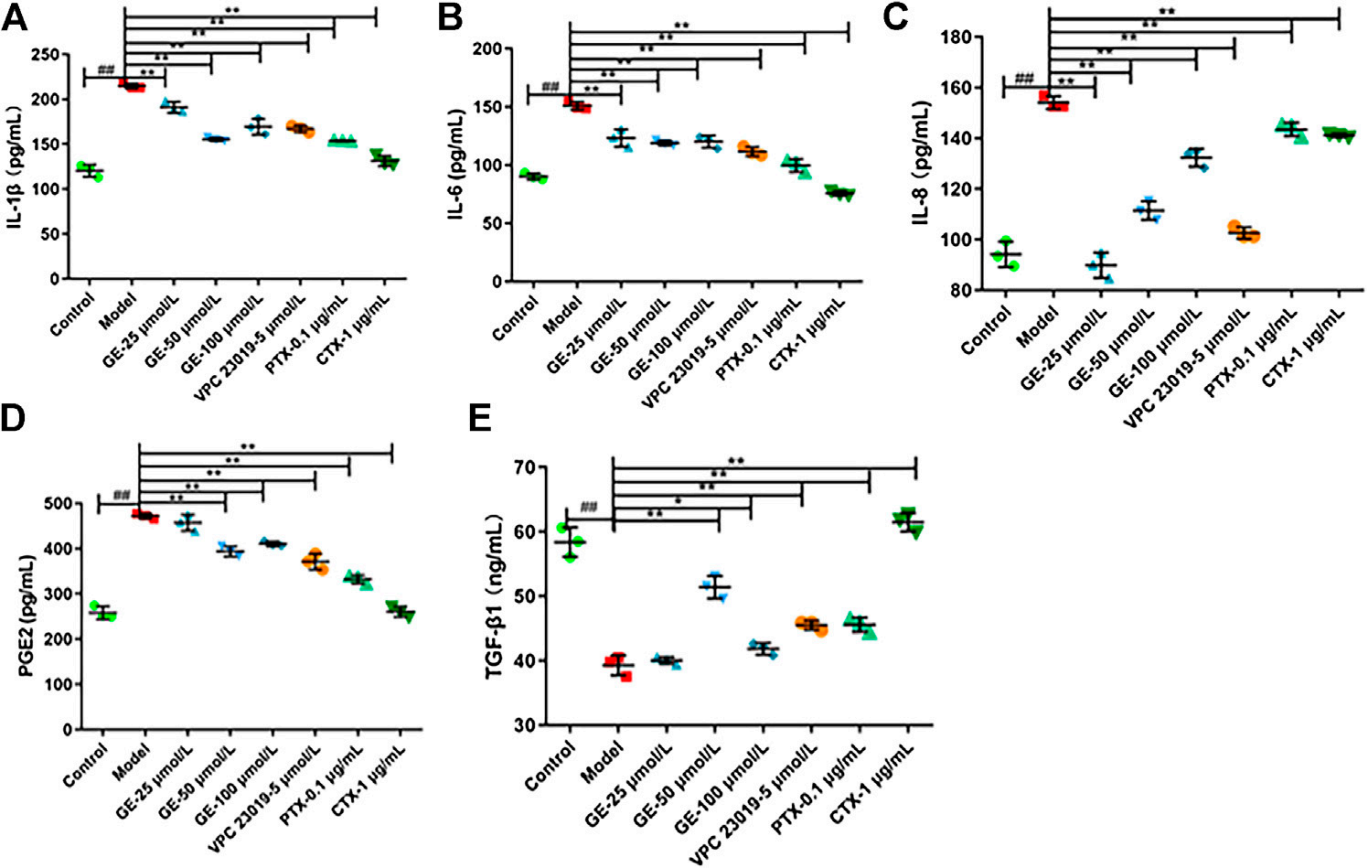
Effect of GE on modulate cytokine secretion in MH7A induced by S1P. MH7A cells were treated with S1P (5 μmol/L) with or without different inhibitors Gαs activator CTX (1 μg/mL), Gαi inhibitor PTX (0.1 μg/mL), S1PR1/3 inhibitor VPC 23019 (5 μmol/L) and GE (25, 50 and 100 μmol/L) for 24 h. Expression of **(A)** IL-1β, **(B)** IL-6, **(C)** IL-8, **(D)** PGE2 and **(E)** TGF-β1 in MH7A was determined by ELISA analysis (n = 3). Data are represented as mean ± SEM. ^*^ *p* < 0.05, ^**^ *p* < 0.01 versus control group; ^#^ *p* < 0.05, ^##^ *p* < 0.01 versus S1P (5 μmol/L) group.

To further confirm the anti-inflammatory effects of GE, MH7A cells were treated with GE, S1PR1/3 inhibitor, Gαi inhibitor and Gαs activator, and then the pro-inflammatory factors IL-1β, IL-6, IL-8, PGE2, anti-inflammatory factor TGF-β1 were detected by using the ELISA kits. As expected, after GE administration, the secretion levels of pro-inflammatory factors IL-1β, IL-6, IL-8, and PGE2 were significantly reduced and the secretion level of anti-inflammatory factor TGF-β1 were significantly increased compared with the model group induced by S1P. Moreover, the higher the dose of GE, the more significant the effect was. Additionally, under the action of inhibitors VPC 23019, PTX, and activator CTX, the levels of anti-inflammatory factor were increased and pro-inflammatory factors were reduced (Figure 6). These results demonstrated that anti-inflammatory effect of GE was associated with S1PR1/3 coupled Gαi/Gαs conversion again.

### Geniposide Regulated Sphingosine-1-Phosphate Receptors1/3 Coupled Gαi/Gαs Downstream Related Proteins Expression in MH7A Cells Induced by S1P

**FIGURE 7:**
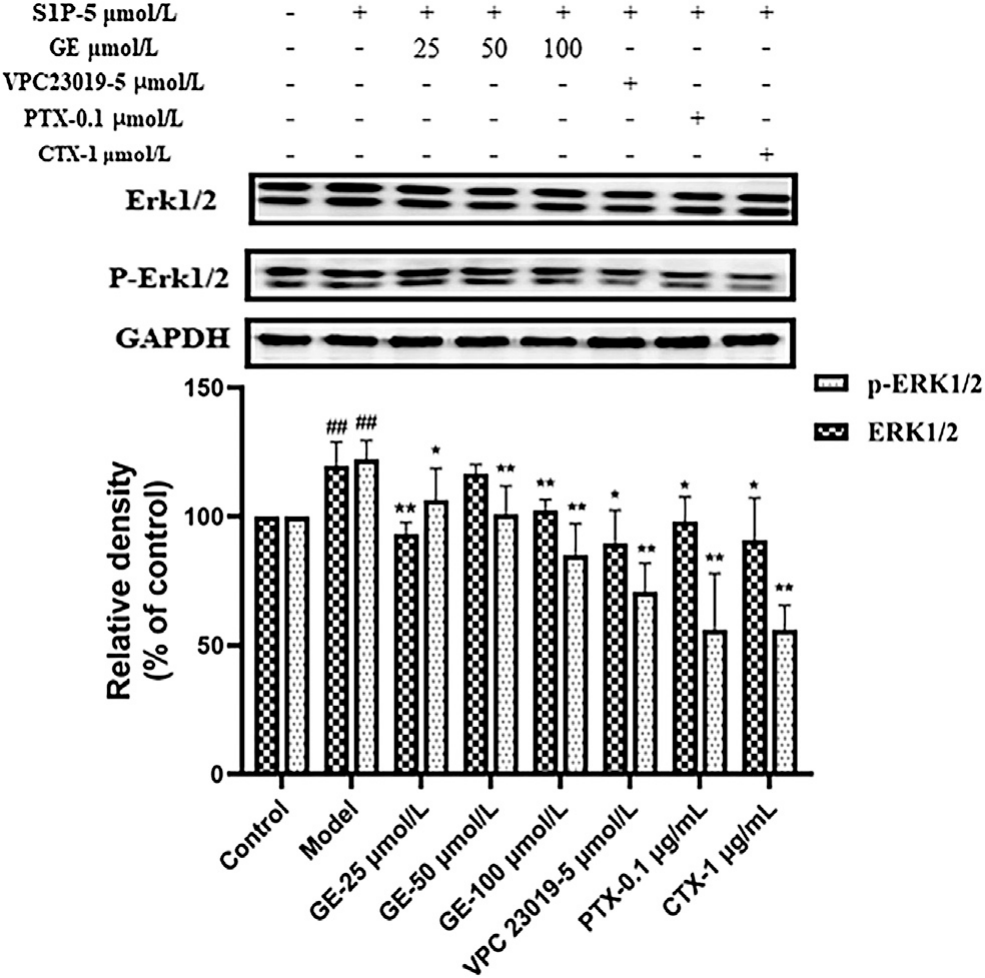
Effect of GE on ERK1/2 in S1P-stimulated MH7A. MH7A cells were treated with S1P (5 μmol/L) with or without different inhibitors Gαs activator CTX (1 μg/mL), Gαi inhibitor PTX (0.1 μg/mL), S1PR1/3 inhibitor VPC 23019 (5 μmol/L) and GE (25, 50 and 100 μmol/L) for 24 h. Western blot analysis of ERK1/2 and *p*-ERK1/2 in MH7A cells (n = 3). Data are represented as mean ± SEM. ^*^ *p* < 0.05, ^**^ *p* < 0.01 versus control group; ^#^ *p* < 0.05, ^##^ *p* < 0.01 versus S1P (5 μmol/L) group.

As demonstrated previously, it had been verified that GE could inhibit the proteins expression of S1PR1/3 and Gαi/Gαs. To further investigate whether the inhibitory effect of GE on ERKl/2-mediated signaling pathway is related to S1PR1/3-Gαi/Gαs, we detected the expression levels of ERKl/2 and *p*-ERK1/2. As seen in Figure 7, the treatment of the cells with GE, inhibitors and activator down-regulated the expression of ERK and *p*-ERK1/2 respectively (*p* < 0.05). The results indicated that GE could inhibit downstream ERK1/2 signaling pathway by downregulating the expression of the S1PR1/3 coupled Gαi/Gαs conversion.

**FIGURE 8:**
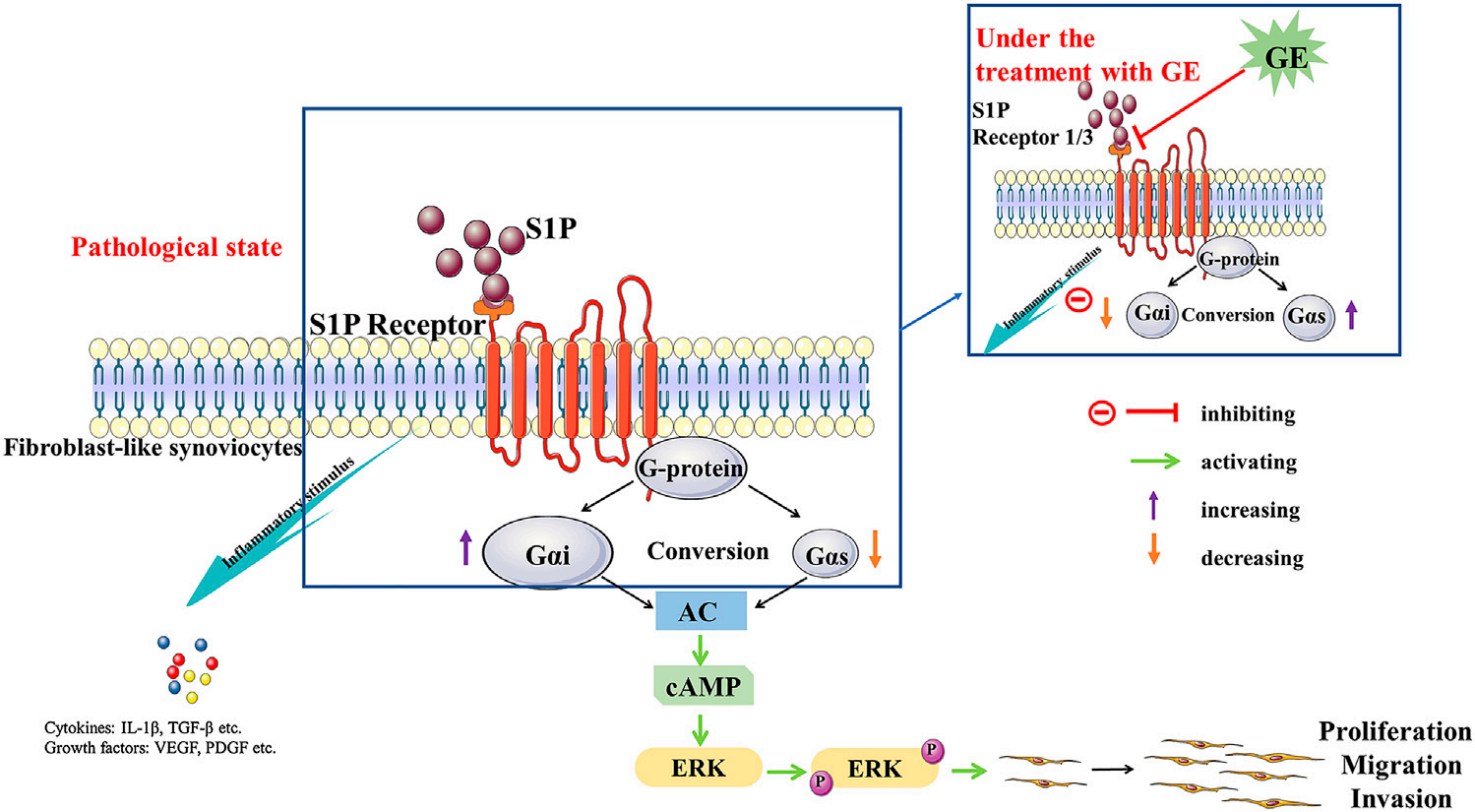
The potential mechanism of cells dysfunctions that S1P may stimulus S1PR1/3 coupled Gαi/Gαs conversion and the regulation of GE. S1P binds to the ligand S1PR1/3, resulting in an imbalance in the conversion of Gαi and Gαs and inducing an inflammatory response. Meanwhile, it mediates the activation of the downstream signal pathway AC/cAMP and protein ERK1/2, which leads to the dysfunctions of FLSs. GE exerts regulation by blocking S1PR1/3 coupling Gαi/Gαs conversion, restoring the dynamic balance of Gαi and Gαs protein and anti-/pro-inflammatory factors.

## Discussion

RASFs are derived from the synovial cells of patients with RA and they are the main effector cells in RA. Moreover, the changes of cells physiological functions, such as abnormal proliferation, migration and invasion are the central events in RA (Liu et al., 2017; Liu et al., 2017; Du et al., 2018). Hyperplasia synovial cells can secrete a large number of inflammatory factors, causing inflammatory cell infiltration hypoxia and synovial microenvironment imbalance (Lubberts, 2015; Tan et al., 2017), ultimately, aggravating the course of RA. Therefore, MH7A cells were used in this experiment. It has been reported that inflammatory factors including S1P, TNF-α, and IL-1β contribute to synovial cells hyperplasia and inflammatory damage of RA. Previously study have shown that S1P, as an important inflammatory mediator of RA, has a higher concentration in synovial fluid than that in the blood. In the present study, we confirmed that S1P can promote inflammation and abnormal proliferation of RASFs, and we further determined the optimum concentration and time of S1P induction. Meanwhile, we found that S1P can cause proliferation, migration and invasion of the cells. While GE at different concentrations (25, 50 and 100 μmol/L) had an inhibitory effect on abnormal physiological functions of RASFs. These results support the view that GE, serves as the active ingredient of *Gardenia jasminoides Ellis*, has the positive effects of RASFs induced by S1P.

Through binding to S1PRs, S1P will destroy the dynamic balance of pro/anti-inflammatory factors, lead to infiltration of inflammatory cells and aggravate the pathological process of RA (Masuko et al., 2012). S1PRs belong to the GPCRs, at present, the role of S1PRs in RA is mainly focused on the studies of its downstream inflammatory signaling pathways (Masuko et al., 2007; Jin et al., 2016). While, the coupling of S1PRs with specific G protein subunits is rarely reported. As the member of the family of S1PRs, S1PR1/3 are mainly involved in the inflammatory response, promoting cell angiogenesis, differentiation and proliferation (Zhao et al., 2008; Inoue et al., 2019). In addition, the transformation of G protein subunit Gαi and Gαs play a vital role in the proliferation functions of RASFs. To further explored the positive effects of GE on RASFs and whether the detailed mechanisms were related to S1PR1/3 and Gαi/Gαs, we used the S1PR1/3 inhibitor VPC 23019, the Gαi inhibitor PTX and the Gαs activator CTX in the S1P-induced cell model. The results showed that these inhibitors, activator could inhibit abnormal proliferation, migration and invasion of the cells. Hence, we had reasons to deduce that inhibiting S1PR1/3-Gαi/Gαs pathway may become a new target for RA treatment. Indeed, GE could regulate cells dysfunctions induced by S1P through inhibiting S1P1/3-Gαi/Gαs transformation and this notion was further supported by the experiments of protein electrophoresis and qRT-PCR. Also, under pathological state, the expression of Gαi was up-regulated, conversely, the expression of Gαs was decreased. Above all, GE could inhibit the abnormal conversion of Gαi/Gαs and restore the balance of them. Notably, it is reported that cAMP, as a second messenger, participates in a variety of inflammatory responses (Jia et al., 2014; Raker et al., 2016). The Gα subunit regulates the downstream signaling pathway through the AC/cAMP signal axis, and Gαi/Gαs regulates the abnormal proliferation of RASFs by mediating the secretion of cAMP (de Castro Abrantes et al., 2019). Additionally, S1P/S1PRs can regulate intracellular cAMP levels, mediate downstream signaling pathways, regulate cell biological functions and aggravate abnormal proliferation of FLSs (Bourgoin et al., 2012). Therefore, cAMP may be an intermediate of S1P-mediated synovial inflammation. In this study, GE was shown to increase the expression level of cAMP. Meanwhile, the inhibitors and activator could also promote the expression of cAMP. Further studies were clarified the co-expression of S1PR1/3 and Gαi/Gαs proteins. S1PR1 and S1PR3 could be co-expressed with Gαi, Gαs respectively and this co-expression ability could be weakened by GE. Thus, these results will contribute to a better understanding of S1PR1/3 coupling Gαi/Gαs conversion and the inhibitory effect of GE on the S1PR1/3-Gαi/Gαs signal axis.

Inflammation is a primary characteristic of RA, which is mainly manifested by the infiltration of inflammatory cells and the release of inflammatory factors (Sweeney and Firestein, 2004). In the normal body, pro-inflammatory and anti-inflammatory factors maintain a dynamic balance and keep the body's normal physiological functions, while, this dynamic balance is broken in RA. During the development of RA, the secretion of pro-inflammatory factors IL-1β, IL-6 and IL-8 were increased (Li et al., 2018). And the factor PGE2 also plays an important role in promoting the development of RA (Afzali et al., 2018; Han et al., 2020). Conversely, the expression of TGF-β1 in RA patients is suppressed. It is reported that silence the expression of TGF-β1 not only induces an inflammatory response, but also promotes migration and invasion of RASFs (Zhu et al., 2019). The levels of IL-1β, IL-6, IL-8, PGE2, and TGF-β1 were evaluated in this study to investigate whether the imbalance of pro-/anti-inflammatory cytokines was associated with inflammatory injury, and the anti-inflammatory effect of GE were explored. Pro-inflammatory factors IL-1β, IL-6, IL-8, and PGE2 secretion levels were increased significantly under the induction of S1P, while the anti-inflammatory factor TGF-β1 secretion level was decreased. Indeed, GE could restore dynamic balance of pro-/anti-inflammatory factors. The treatment with inhibitors VPC 23019, PTX and activator CTX had the same effects as GE. These results indicate that GE plays a role in immune regulation by restoring the dynamic balance between pro-inflammatory cytokines and anti-inflammatory cytokines and that is related to the S1PR1/3-Gαi/Gαs signaling pathway.

It has been evaluated that the protein ERK1/2 can activate different signal transduction pathways, which are related to cell growth, proliferation, differentiation and survival (de Jong et al., 2016). In addition, ERK1/2 exists in synovial tissues and participates in signal transduction of various extracellular stimuli (Namba et al., 2017). The abnormally activated ERK1/2 is closely related to the proliferation of RASFs and the pathological process of joint destruction (Jiang et al., 2017). Studies have shown that S1P relate to the MAPKs signaling pathway closely and mediates changes in cells physiological functions (Sato et al., 2012). In this study, the expression of ERK1/2 and *p*-ERK1/2 proteins were analyzed. The results showed that the expression of *p*-ERK1/2 was up-regulated under the induction of S1P, and this abnormal activation was suppressed after the treatment of GE. Meanwhile, the inhibitors VPC 23019, PTX and activator CTX had the same effects as GE. Therefore, we have demonstrated that GE can reduce the inflammation and abnormal physiological functions of RASFs by regulating the expression of S1PR1/3 coupled Gαi/Gαs conversion. To summarized, GE is similar to S1PR1/3 inhibitor VPC 23019, which can inhibit S1P binds to S1PR1/3 in RASFs and recover the conversion balance of Gαi and Gαs. Thereby, GE reduces the abnormal proliferation, migration and invasion of the RASFs so that it can alleviate the pathological manifestations of RA, and thus it playing a therapeutic role.

## Conclusion

In general, this study indicates that through binding to S1PR1/3, S1P leads to the abnormal conversion of Gαi/Gαs and destroys the dynamic balance of anti-/pro-inflammatory factors as well. This experiment has highlighted that GE can alleviate the S1P-stimulated dysfunctions of MH7A cells and block the activation of the downstream ERKl/2-mediated signaling pathway by inhibiting S1PR1/3 mediating the conversion of Gαi and Gαs. Thereby, GE restores the dynamic balance of Gαi and Gαs indirectly. This experiment has only done *in vitro*, and the further study is required to research *in vivo*. Finally, our data provide new ideas into detail mechanisms underlying the anti-inflammatory and immune effects of GE and the S1PR1/3-Gαi/Gαs pathway may be a helpful target for RA therapy.

## Data Availability Statement

The raw data supporting the conclusions of this article will be made available by the authors, without undue reservation.

## Author Contributions

Participated in research design: RW, XD, and HW. Conducted the experiments: RW, XD, MW, RD, YW, and YB. Contributed new reagents or analytic tools: MS and HZ. Performed the data analysis: RW and XD. Wrote or contributed to the writing of the manuscript: RW.

## Funding

This work was supported by grants from the National Natural Science Foundation of China (Nos. 81874360, 81473400, and 81073122).

## Conflict of Interest

The authors declare that the research was conducted in the absence of any commercial or financial relationships that could be construed as a potential conflict of interest.
